# The Pathogenesis, Clinical Features, and Treatment of *Corynebacterium striatum*-Related Infection

**DOI:** 10.3390/microorganisms14010119

**Published:** 2026-01-06

**Authors:** Huan Zhang, Zheng Zhang, Haiqing Shi, Jianbo Li, Xuelian Liao

**Affiliations:** 1 Department of Critical Care Medicine, West China Hospital, Sichuan University, 37 Guo Xue Xiang Street, Chengdu 610041, China; zhuan@wchscu.cn (H.Z.); zhangzhengicu@stu.scu.edu.cn (Z.Z.); shihaiqing@stu.scu.edu.cn (H.S.); lijianbo@scu.edu.cn (J.L.); 2 Department of Critical Care Medicine, West China Tianfu Hospital, Sichuan University, Chengdu 610218, China

**Keywords:** *Corynebacterium striatum*, pathogenesis, multidrug-resistant, clinical features, treatment

## Abstract

Purpose of review: *Corynebacterium* *striatum* (*C. striatum*) has rapidly evolved into a virulent, multidrug-resistant pathogen in recent years. This review aims to summarize the pathogenesis, clinical characteristics, and treatment strategies associated with *C. striatum*-related infection. Recent findings: The recent advances in epidemiology, newly identified virulence and resistance determinants are highlighted. Therapeutic failure in affected patients has been associated with poor, and sometimes fatal, outcomes. Notably, clinical manifestations and therapeutic approaches vary depending on the site of infection. Potential therapeutic targets (including novel promising antibacterial agents, and combination therapy approaches), and the clinical characteristics associated with *C. striatum* infection are summarized. Summary: Given the rapid evolution of *C. striatum*, it is particularly important to recognize that clinical features and optimal treatment strategies may differ by infection site. Further research is needed to elucidate its clinical and genetic characteristics.

## 1. Introduction

**Figure 1:**
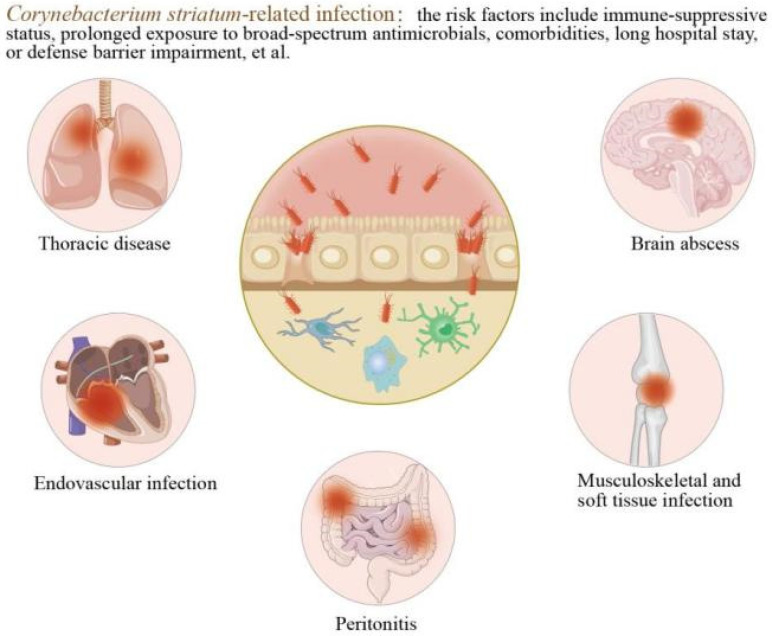
*Corynebacterium striatum*-related infections and their risk factors (created by the authors).

As a gram-positive, aerobic and facultatively anaerobic, and non-motile *Bacillus*, *Corynebacterium striatum* (*C. striatum*) was previously regarded as a commensal organism and a normal constituent of the skin and mucosal flora. However, its apparent virulence has evolved rapidly, and multidrug-resistant (MDR) strains are now increasingly reported worldwide [1]. Severe and sometimes fatal infections caused by this bacterium have been described with growing frequency, particularly among patients with chronic comorbidities, prolonged hospitalization, immunosuppression, extended exposure to broad-spectrum antimicrobials, or impaired barrier defenses, and have occasionally led to nosocomial outbreaks, as shown in Figure 1. Notably, an increasing number of cases have also been reported in immunocompetent individuals across all age groups, in some instances with catastrophic outcomes [2].

Some infections have resulted in fatal outcomes and are associated with high mortality rates [3]. As reported, the mortality rates of *C. striatum* bacteremia and *C. striatum*-related pneumonia have reached 34% and 49%, respectively [4,5]. Given these high mortality rates, greater clinical attention to *C. striatum* infections appears warranted.

Even with the implementation of antimicrobial stewardship programs, the incidence of multidrug-resistant (MDR) microorganisms in intensive care units (ICU) remains high (for example, the production of extended-spectrum β-lactamases by gram-negative bacteria is as high as 72%) [6]. *C. striatum* strains, especially MDR clones, pose a significant challenge for infection control in both ICUs and general wards. Despite its growing clinical significance, comprehensive reviews that synthesize current knowledge on *C. striatum*—encompassing its pathogenesis, resistance mechanisms, clinical features and management strategies—remain limited. Therefore, it is imperative to highlight and summarize the emerging and clinically significant features of *C. striatum.*

## 2. History and Current Trend

In 1976, the first case of *C. striatum*-related pleuropulmonary infection was reported in a patient with chronic lymphocytic leukemia and resulted in a fatal outcome [7]. The first nosocomial outbreak of *C. striatum* was described in 2007 among patients with chronic obstructive pulmonary disease (COPD), and all isolates were identified as multidrug-resistant [8]. The detection of *C. striatum* infection, particularly the MDR strains, has increased globally and is often associated with nosocomial settings, in intensive care units (ICUs), and the use of invasive medical devices [3]. A recent survey from South Korea indicated that the proportion of *C. striatum*-infected critical hospital-acquired pneumonia (HAP) increased from 1.0% during 2014–2015 to 5.4% during 2018–2019 [9]. In Europe, China, and other regions, hospital outbreaks and nosocomial transmission have been reported much more frequently, primarily through contaminated surfaces, healthcare worker contact, or shared medical equipment [1,10]. Molecular tracing has shown that these outbreaks often involve a single clone and are accompanied by the dissemination of resistance genes such as *ermX*, *tet*, and others [10]. With regard to the *C. striatum*-related lower respiratory tract infections, most cases are hospital-acquired, including those associated with mechanical ventilation, while community-acquired infections remain uncommon [9].

## 3. Coinfection with Other Bacteria, Fungi, and Viruses

Reports of *C. striatum* co-infection with other bacteria, fungi, and viruses are not uncommon, including *Acinetobacter baumannii*, *Aspergillus* spp., or *Pneumocystis jirovecii* [11]. Polymicrobial infection involving other bacteria species has gained increasing attention in recent years, particularly in respiratory, wound, and bloodstream infections. Among patients with influenza, *C. striatum* has been identified as a major contributor to super-dominant secondary bacterial infections (SBIs) [12]. During the COVID-19 pandemic, the incidence of SBIs associated with multidrug-resistant *C. striatum* increased substantially. The antimicrobial resistance profile of this organism continues to evolve, raising significant concern, and even linezolid resistance has been reported [13].

When co-existing with other bacterial species, *C. striatum* may influence their virulence, leading to either attenuation or enhancement. Recent research progress and relevant clinical features are summarized in Table 1. The following sections outline several proposed mechanisms underlying these symbiotic interactions.

(1) Biofilm Synergy: *C. striatum* can form multilayered biofilms on various material surfaces, including polyurethane and silicone. When coexisting with *Staphylococcus aureus*, bacteria may mutually enhance biofilm structural stability and increase antibiotic tolerance. Such synergistic interactions are strongly associated with the persistence of chronic infections and their therapeutic challenges [14,15].
(2) Under selective pressure from broad-spectrum antibiotic therapy, susceptible bacteria are suppressed, creating an ecological niche that allows antibiotic-resistant organisms such as *C. striatum* to emerge as secondary dominant species [10]. Consequently, in patients receiving prolonged antimicrobial therapy, *C. striatum* frequently acts as a secondary pathogen within polymicrobial infections [16].
(3) Immunosuppression and device dependence: Conditions such as prolonged mechanical ventilation, indwelling medical devices, dialysis, and chemotherapy for malignancy create environments that are highly conducive to biofilm formation and microbial colonization. Patients with these risk factors exhibit a significantly higher incidence of polymicrobial infections than the general hospitalized population.
(4) Additional microbial interactions: Certain bacterial or fungal species may exert antagonistic activity against *C. striatum*, including tuberculosis and Corticoid Fungi [17,18].

**Table 1:** Co-infection of *C. striatum* with Other Bacteria: Research Progress and Clinical features.

Site of Infection	Predominant Co-Pathogens	Clinical Features
Respiratory tract: such as pneumonia or ventilator-associated infections [10]	*Staphylococcus aureus*, *Pseudomonas aeruginosa*, *Acinetobacter baumannii*, *Klebsiella pneumoniae*, *Stenotrophomonas maltophilia*, *Enterobacter cloacae*.	The most common site of co-infection, typically observed in ICU patients. *C. striatum* often emerges as a secondary pathogen or even replaces the initially dominant microorganism.
Bloodstream infection [1,19]	*Staphylococcus epidermidis*, *Enterococcus* spp., *Candida* spp.	Commonly seen in catheter-related bloodstream infections (CRBSIs); *C. striatum* often contaminates catheters together with skin-colonizing bacteria and leads to true infection.
Wounds/surgical wounds/burn sites [20]	*S. aureus*, *Enterococcus faecalis*, *Proteus mirabilis*, *E. coli*.	*C. striatum* can form biofilms together with Gram-negative bacilli, exacerbating wound infections.
Urinary tract or implanted device infections [2,21]	*Enterococcus faecium*, *E. coli*, *Klebsiella* spp.	Relatively uncommon, usually associated with urinary catheters or implanted devices.

Significant regional variation has been reported in co-infection rates: the highest rates (>50%) occur in respiratory tract infections, particularly in ICU settings [22], whereas bloodstream infections exhibit comparatively lower rates (approximately 20–30%) [19]. *C. striatum* may contribute synergistically to pathogenicity and antimicrobial resistance within polymicrobial infections and should not be regarded merely as a contaminant. Patients with polymicrobial infections may experience prolonged mechanical ventilation, greater difficulty in infection control, and higher mortality [1,23].

## 4. Recent Identification of *C. striatum* in Clinical and Laboratory

Conventional bacterial culture remains the preferred method for initial screening of *C. striatum,* as it is accessible, cost-effective, and allows recovery of viable isolates for subsequent identification and antimicrobial susceptibility testing. However, colony morphology is highly variable and may be easily confused with other coryneform bacteria.

Currently, matrix-assisted laser desorption/ionization time-of-flight mass spectrometry (MALDI-TOF MS), which identifies organisms based on protein profile, has become a cornerstone for the rapid and accurate identification of *C. striatum* depending on mass-spectra database [24]. When database completeness is insufficient, 16S or rpoB sequencing is still required for confirmation [24], and these approaches remain among the “gold standards” for *C. striatum* identification.

High-resolution melting (HRM) analysis and other rapid molecular methods have been developed for the direct detection of *C. striatum* from specimens such as sputum, serving as complementary tools to culture and significantly reducing diagnostic turnaround time [25]. When rapid differentiation from related *Corynebacterium* species (e.g., *C. propinquum*) is required, HRM and species-specific PCR appear to hold promising clinical potential.

Other novel identification approaches, such as targeting the ftr1 gene for *C. striatum*-MCDA-VR assay and the ssrA gene for qPCR detection, have been verified as rapid and cost-effective methods for identifying *C. striatum* [19,23,24,25,26].

With the increasing prevalence of MDR strains, broth microdilution (BMD) and commercial microdilution panels have become the recommended susceptibility testing methods [27]. However, for Corynebacterium spp., interpretive breakpoints are not uniformly established by CLSI or EUCAST across all antibiotics. Ongoing discussions and standardization efforts related to CLSI document M45, which addresses testing of infrequently isolated or fastidious Gram-positive bacilli, are helping to promote greater harmonization in this area. Biofilm-forming ability of *C. striatum* contributes to its persistence and antimicrobial resistance [28]. Biofilm-related assays and in vitro biofilm susceptibility testing have been used in research settings to better explain clinical treatment challenges, although these methods have not yet been incorporated into routine clinical diagnostics [29].

## 5. Molecular Subtype of *C. striatum*

Molecular typing of *C*. *striatum* has important applications in clinical practice and infection control. including outbreak tracing, understanding the dissemination of resistance genes among different clones, and supporting surveillance efforts. Common molecular typing approaches include the following: (1) Pulsed-Field Gel Electrophoresis (PFGE): Widely used in early epidemiological studies to characterize clonal dissemination within hospitals [10,30]. (2) Multilocus Sequence Typing (MLST): Applied to define sequence types (STs) and clonal complexes (CCs) [31]. (3) cgMLST/WGS-SNP/Whole-Genome Sequencing (WGS): Currently considered the highest-resolution and most reliable methods for intra- and inter-hospital outbreak investigation. Also, cgMLST and core-SNP analyses appear suitable for large-scale surveillance [10]. (4) Single-Gene or Partial-Gene Sequencing (e.g., *rpoB*, *gyrA*, *16SrRNA*): Provides lower resolution but serves as a useful supplementary identification tool when resources are limited [19,32].

Due to variations in study populations, infection control, sampling strategies, and detection methods, the predominant sequence types (STs) and clone groups (CGs) of *C. striatum* isolates show differences across different regions. In the study by Jin Woong Suh et al. [22], the most common STs in the Republic of Korea were ST2, ST3, ST6, and ST5. Margarita Gomila et al. reported that ST2, ST4, ST1, and ST11 were the most prevalent sequence types in Spain [23]. In China, *C. striatum* also showed high genetic diversity, with CC19 identified as the predominant clonal complex, while ST16 within this complex has been detected most frequently. CG4, CG5, CG26, CG28, and CG55 have been considered potentially hypervirulent and multidrug-resistant [24]. Two related MDR clones identified by PFGE indicated that PFGE type I was most common in ICUs, surgical wards, and among patients with hematogenic infections [19,30].

## 6. Virulent Agents

The genes involved in multiple biological functions in *C. striatum* (the main agents are shown in Table 2) include those related to adherence and biofilm formation, iron uptake and metabolism, stress responses, molecular chaperone activity (e.g., *groEL*), intracellular survival (e.g., *sodA*), and the two-component regulatory system (*regX3*). The team of Du, L employed whole-genome sequencing to construct a single-nucleotide polymorphism (SNP)-based phylogenetic tree of 27 *C. striatum* clinical isolates, which exhibited varying degrees of cytotoxicity. The genes *humU, irp6B, regX3, groEL, sigA, sodA*, and *sigH* were present in all tested isolates. In contrast, *spaE, spaF, spaD, srtB*, and *srtC* were detected in most isolates and are associated with adhesion [19]. Additional adherence-related virulence determinants include pilus proteins encoded by the spa operon.

A key characteristic of *C. striatum* is its ability to form biofilms, which is considered a major virulence factor that may facilitate nosocomial transmission and persistence in clinical settings. Biofilm formation also appears to enhance multidrug resistance and immune evasion of *C. striatum* strains [33]. All *C. striatum* isolates with strong biofilm-forming capacity expressed spaDEF, whereas those with moderate or weak biofilm formation did not [34]. The extracellular matrix has been shown to be essential for biofilm formation in both general and MDR *C. striatum* isolates [28,35].

**Table 2:** Research Progress on Main Virulence Factors of *C. striatum*.

Category	Representative Genes/Proteins	Function/Mechanism	Notes/Evidence
Adhesion and colonization [33]	*srtA*, *spaA–F*, LPXTG-motif adhesins	Surface anchoring and epithelial adhesion	Promote colonization on skin and medical devices
Biofilm formation [33]	spaD, spaE, spaF, spaG, spaH, spaI, *srtA,* srtB, srtC, srtD, strE, exopolysaccharide genes	Biofilm maturation and persistence	Increased antibiotic tolerance in biofilm state
Cell wall and lipid metabolism [35]	*pks*, *accD*, *mmpL*, *fadD*	Mycolic acid synthesis; cell envelope integrity	Enhances resistance to host defenses
Iron acquisition systems [36]	fagA, fagB, fagC, fagD, humU, irp6A, irp6B, *fetA*, *fetB*, siderophore transporter genes	Iron uptake in host environment	Facilitates intracellular survival
Oxidative stress and immune evasion [37]	sigA, sigH, *sodA*, *katA*, *groEL*, *dnaK*	Detoxification of reactive oxygen species	Promotes persistence under immune stress
Secreted enzymes/potential toxins [38]	*rpf* *A* *, rpf* *B* *, rpf* *I*	Tissue damage and inflammation	Non-diphtherial but contributes to virulence

## 7. Multidrug Resistance

MDR *C. striatum* isolates commonly exhibit resistance to a broad spectrum of antibiotics directed against Gram-positive bacteria. The major categories of antimicrobial resistance mechanisms in *C. striatum* are summarized in Table 3. Similar resistance profiles have been reported in both infection-related and colonizing or contaminant clones, with minimal variation in antimicrobial susceptibility [39].

Antimicrobial resistance in *C. striatum* may arise through two distinct mechanisms: endogenous and exogenous. The former typically involves chromosomal mutations, as described for fluoroquinolones and daptomycin. In contrast, resistance to macrolides, tetracyclines, phenicols, β-lactams, and aminoglycosides is largely attributed to exogenous resistance genes carried by mobile genetic elements, including insertion sequences (ISs), plasmids, transposons, and bacteriophages [40].

(1) Endogenous multidrug resistance

Resistance to quinolones is mainly caused by mutations in the *gyrA* and *parC* genes. In *C. striatum*, two common mutations in codons 87 and 91, along with four novel mutations in the *gyrA*, have been identified as contributors to the quinolone resistance [41]. In contrast, the *parC* gene has not been successfully amplified in reported studies.

Daptomycin possesses potent activity against Gram-positive bacteria; however, even short-term exposure may lead to high-level daptomycin resistance (HLDR) in *C. striatum* [46]. A loss-of-function mutation in *pgsA2*, such as an IS30 insertion that disrupts *pgsA2*, has been shown to be necessary and sufficient for the development of HLDR [47].

(2) Exogenous Multidrug Resistance

Exogenous multidrug resistance in *C. striatum* is mediated by a diverse set of mobilizable resistance genes, which may promote bacterial proliferation and contribute to outbreaks that are difficult to control. These mobilizable genetic elements include integrative and conjugative elements, plasmids, insertion sequences, transposons, prophages, integrons, and genomic islands.

Notably, the presence of resistant genes does not always translate into phenotypic resistance. For example, one *C. striatum* strain remained susceptible despite containing the erm(X) gene [48]. Similarly, another isolate carrying the vancomycin-resistant gene *vanW* was verified to be susceptible to vancomycin [44].

(2.1) Insertion Sequences

Insertion Sequences (ISs) are small, simple, and autonomous mobile genetic elements that are widely distributed. In *C. striatum*, ISs, mainly belonging to the IS3 and IS256 families [41], are believed to contribute to the development of antibacterial resistance and virulence.

Several chromosomal genes, including *dppD* and *cgrA/B*, have been identified as hotspots for the insertion of mobile elements that may carry complex ISs and multiple integrases. Moreover, antimicrobial resistance genes such as *erm(X)*, *aac(3)-XI*, and *tet(W)* have been reported in association with the insertion sequences IS1249, ISCg9a, and IS3504, respectively [49].

(2.2) Plasmids

Plasmid-borne resistance genes have been detected in *C. striatum*. In Catherine Urrutia’s study [21], plasmid pJA144188 carried six resistance genes (ErmX, tet(W), cmx, sul1, aadA, aac(6′)-Ia), whereas plasmid pTP10 harbors five (ErmX, tetA, cmx, aph(6)-Id, aph(3′)-Ia). An additional unnamed plasmid contained only a single resistance gene, ErmX.

(2.3) CRISPR-Cas

Cas proteins are products of bacterial CRISPR-Cas systems and facilitate the degradation of foreign nucleic acids, including phages and plasmids. The CRISPR-Cas system appears to show considerable diversity in *C. striatum*. In an analysis of 10 genomes from multidrug-resistant *C. striatum* clinical isolates collected at a public hospital in Rio de Janeiro, Brazil, type I-E gene arrangements were identified, along with three additional multidrug-resistant isolates, and alternative type I-E gene arrangements were identified [50]. Moreover, most CRISPR spacers in *C. striatum* clinical isolates are uncharacterized indicating that there is a substantial reservoir of unexplored corynebacteriophages and plasmids.

## 8. Clinical Features

The prevalence of *C. striatum*-associated diseases appears to be increasing. Reported conditions include endovascular infection, thoracic disease, musculoskeletal disease, soft tissue infection, abdominal disease, fatal brain abscesses [51], nosocomial urethritis [52], tubo-ovarian abscess [53], and others. These clinical presentations are summarized in Table 4.

(1) Endovascular infection

*C. striatum*-related endovascular infections appear in multiple forms, including native or prosthetic valve endocarditis, implantable device infection, thrombophlebitis, arteritis, and even artery rupture. Immune suppression, underlying comorbidities, and cardiovascular implantable devices may facilitate infection. *C. striatum* endocarditis has also been reported in patients without anatomic changes and immunosuppression [56]. The related clinical presentations are generally nonspecific, including arthralgia, back pain, fevers, chills, fatigue, sweats, dizziness, etc. Although *C. striatum* is often dismissed as a contaminant, it may cause true native valve endocarditis and warrants clinical consideration when clinical features are compatible. Also, the presentation of angiopathy may be confused by autoimmune vasculitis of non-infectious etiology, or the blood culture *C. striatum* as contaminants may be ignored. Prognosis in adults appears poor, with high mortality, particularly in patients with polyinfection, renal dysfunction, and absence of a central venous catheter [57]. By contrast, reported infections in children tend to present with milder symptoms and lower mortality [58].

(2) Thoracic disease

*C. striatum*-related thoracic diseases present not only as pneumonia but also as intrapulmonary abscess, multiple pulmonary nodules, and even life-threatening mediastinitis. Patients with pneumonia often have severe or chronic underlying respiratory conditions that require frequent and prolonged hospitalizations, exposure to invasive procedures or immunosuppressive therapy. *C. striatum* infection may induce pronounced lung inflammation and pathological alterations, characterized by enhanced neutrophilic infiltration and significant upregulation of pro-inflammatory mediators, including interleukin (IL)-17, IL-6, and CXCL-8, accompanied by activation of the NF-κB signaling pathway [59].

Positive *C. striatum* cultures have been obtained from a wide range of respiratory specimens, and several outbreaks have been reported worldwide. Clinical outcomes in many affected patients have been poor. The clinical improvement of targeted antibiotic therapy against *C. striatum* in the lower respiratory tract may be limited [60]. The high mortality rates may be partly attributed to the underlying severity of respiratory disease, as well as to the aggravating effect of *C. striatum* infection [5].

(3) Musculoskeletal and soft tissue infection

The aggressive strains of *C. striatum* may infect both immunocompetent and immunocompromised patients, leading to native or prosthetic joint infection (PJI) and, in some cases, osteomyelitis with concurrent empyema. Clinical manifestations are generally nonspecific and typically include swelling, erythema, tenderness, or a reduced range of motion.

Data from the Mayo Clinic Total Joint Registry indicate that *C. striatum* is the most frequently isolated species in monomicrobial *Corynebacterium* PJIs. Infection with *C. striatum* appears to substantially worsen wound severity and may impair healing. Nearly all patients with *Corynebacterium* PJI experience an indolent, chronic disease course characterized by delayed or late-onset infection, most commonly presenting with localized joint pain and swelling. In contrast, systemic symptoms and sinus tract formation are uncommon [54]. The median interval between the most recent arthroplasty and *C. striatum*-associated PJI is typically prolonged, underscoring the importance of biopsy and culture to establish an accurate diagnosis [61].

(4) Abdominal disease

The patients who suffered *C. striatum* peritonitis typically have indwelling peritoneal catheters and underlying chronic renal failure. Clinical symptoms are generally mild and nonspecific. Some patients present without abdominal discomfort or fever, whereas others may experience abdominal pain or pyrexia accompanied by cloudy peritoneal effluent, in which neutrophils predominate on laboratory analysis [62].

Peritonitis associated with *C. striatum* in peritoneal dialysis patients may persist despite appropriate antimicrobial treatment. In such cases, and particularly among patients with recurrent episodes, peritoneal dialysis catheter removal should be strongly considered.

(5) Recurrence

Multidrug resistance and biofilm formation appear to facilitate the recurrence of *C. striatum* infections [7]. The *C. striatum-related* PJIs are associated with high treatment failure rates, including substantial risks of reoperation and reinfection [54]. Recurrence may follow the initial *C. striatum* infection itself or emerge secondary to other bacterial infections. For example, *C. striatum* bacteriemia has been reported to reappear several weeks after apparent resolution of the initial *C. striatum*-related blood infection [63]. Similarly, therapy directed at vertebral osteomyelitis caused by other species may yield favorable outcomes, while the localized *C. striatum* infection subsequently relapses [64].

## 9. Treatment

In general, strict source control (e.g., removal of implant devices or surgery) together with adequate time and doses of antibiotic therapy remains essential for managing *C. striatum*-related disease [65]. Insufficient therapy may increase the bacterial burden with biofilms and facilitate detachment of organisms from the biofilm’s structure to neighboring tissues, even into the bloodstream [14]. Current literature and susceptibility data indicate that vancomycin or linezolid are the most suitable antimicrobial agents. Also, dalbavancin was reported to be another safe and attractive option for *C. striatum*-related infections [66,67,68].

Sometimes *C. striatum* infection is a nerve-racking dilemma for clinicians. When *C. striatum* cultured positively among respiratory infectious samples, true infection is more likely when patients present with compatible respiratory manifestations and new pulmonary infiltrates, accompanied by repeated or predominant isolation of *C. striatum*, particularly in high-risk or immunocompromised individuals. When the clinical condition worsens, it is difficult to confirm whether the severity of the condition was due to respiratory disease, *C. striatum*, or other pathogens’ assault [5]. When monotherapy fails, successful management, especially in polymicrobial or recurrent infections-typically requires targeted antimicrobial combination therapy in conjunction with appropriate source control measures, such as device removal or surgical debridement [19,54]. For adult patients with *Corynebacterium*-related PJI, management generally involves prolonged antibiotic treatment, debridement, and implant retention or surgical treatment [69]. In pediatric settings, antimicrobial therapy may be the preferred initial approach for epiphyseal or metaphyseal infection [70]. In some reported cases, catheter-related *C. striatum* peritonitis has been successfully treated with adequate antimicrobial therapy alone; however, due to frequent relapse and the organism’s biofilm-forming capacity, catheter removal or exchange is often required.

Several novel promising antibacterial strategies are emerging. Enzymatic degradative agents targeting the extracellular matrix, such as proteinase K, dispersin B, and DNase I, have demonstrated strong activity in disrupting biofilm-forming abilities of *C. striatum* [39]. Renewed anti-biofilm approaches, including Antimicrobial peptides (biofilm-AMPs), also show activity specifically directed against biofilm structures [71]. In addition, the anthelmintic drug niclosamide has been reported to degrade the biofilm viability and reduce cell viability drastically with concentration-dependent characteristics [72]. Other potential antibacterial options include bacteriophages targeting MDR *C. striatum*, locally administered lysins, and CXCL10 [73,74,75]. However, further evaluation is needed to determine whether these therapeutic methods are feasible for clinical application.

## 10. Conclusions

Increasing evidence highlights the pathogenic potential of *C. striatum,* particularly multidrug-resistant (MDR) strains. This review summarizes recent advances in epidemiology, newly identified virulence (such as biofilm formation, iron acquisition systems, and other genes contributing to pathogenicity) and resistance determinants (both intrinsic and acquired resistance are elaborated), potential therapeutic targets (including novel promising antibacterial agents, and combination therapy approaches), and the clinical characteristics associated with *C. striatum* infection. Given the rapid evolution and expanding resistance profile of *C. striatum*, further research is warranted to elucidate its clinical relevance and underlying genetic mechanisms.

## Figures and Tables

**Table 3:** Categories of Antimicrobial Resistance Mechanisms in *C. striatum*.

Category of Resistance Mechanism	Molecular Basis/Representative Genes	Affected Antibiotic Class	Mobile Genetic Elements/Genetic Context
Target modification	*erm(X)* (23S rRNA methylation); *gyrA*, *parC* mutations (QRDR)	Macrolides, Lincosamides, Fluoroquinolones	Transposon Tn5432, Chromosomal point mutations [40,41]
Enzymatic inactivation	*aac(3)-XI*, *aph(3′)-Ic*, *ant(4′)-Ib*, *bla*	Aminoglycosides, β-lactams	Plasmid-encoded or IS-associated [42]
Efflux pump-mediated resistance	*tet(W)*, *tetA/B*	Tetracyclines	Integrative conjugative elements (ICEs) [43]
Reduced permeability/cell wall alteration	Cell wall thickening, altered lipid metabolism	Glycopeptides (vancomycin), Daptomycin	Chromosomal regulation, no *van* genes detected [22,44]
Mobile genetic element-mediated dissemination	*erm(X)*–*tet(W)*–*aac(3)-XI* gene clusters	Multiple antibiotic classes (MDR phenotype)	Transposons IS6100, Tn5432, ICEs [45]

**Table 4:** The main clinical characteristics of *C. striatum* infection.

Infection Site	Common Medical History	Usual Clinical Sign	General Outcome
Thoracic infection	structural lung diseases; immunocompromised status;	pneumonia; intrapulmonary abscess; pulmonary nodules; mediastinitis;	In-hospital mortality rates were as high as 70.4% [7]
Endovascular infection	device implantation; immunocompromised status;	native endocarditis; prosthetic implantable device infection; masquerading as a myxoma in atrium; thrombophlebitis; arteritis; artery rupture; bacteremia; sepsis;	mortality rates were as high as 34% [9]
Musculoskeletal and soft tissue infection	arthroplasty surgery; immunocompromised status;	native joint infection; prosthetic joint infection; osteomyelitis; osteoarthritis; tenosynovitis; cellulitis; axillary malodor;	mortality was low; failure was high: reinfection (33%); [54]
peritonitis	catheter indwelling; chronic renal failure;	purulent peritonitis;	doing well
meningitis	unhealed wound; ventricular draining;	purulent meningitis; abscesses	mortality rates were as high as 30% [55]

## Data Availability

No new data were created or analyzed in this study. Data sharing is not applicable to this article.

## Abbreviations

MDR: multidrug-resistant, *C. Striatum*: *Corynebacterium striatum*, ICU: intensive care units, COPD: chronic obstructive pulmonary disease, HAP: hospital-acquired pneumonia, SBIs: super-dominant secondary bacterial infections, STs: sequence types, CGs: clone groups, PFGE: pulsed-field gel electrophoresis, IS: insertion sequences, HLDR: high-level daptomycin resistance, MGEs: mobile genetic elements.
